# Advanced non-small cell lung cancer with SMARCA4 deficiency: a case report

**DOI:** 10.3389/fonc.2026.1901621

**Published:** 2026-08-10

**Authors:** Jingjing Chai, Yingying Gong, Li Gong, Lurun Wang, Xinzhou Deng, Zhiguo Luo, Jian Xiong, Ming Luo

**Affiliations:** 1 Department of Oncology, Taihe Hospital, Hubei University of Medicine, Shiyan, Hubei, China; 2 Shiyan Key Laboratory of Cancer Therapy Resistance and Clinical Translational Study, Shiyan, Hubei, China; 3 Department of Dermatology, Taihe Hospital, Hubei University of Medicine, Shiyan, Hubei, China; 4 Department of Cardiovascular Disease Diagnosis and Treatment Center, Taihe Hospital, Hubei University of Medicine, Shiyan, Hubei, China

**Keywords:** case report, chemotherapy, non-small cell lung cancer, SMARCA4 deletion, targeted therapy

## Abstract

SMARCA4-deficient non-small cell lung cancer (SMARCA4-dNSCLC) is an uncommon malignant epithelial neoplasm originating in the lung, characterized by poorly differentiated and highly invasive pathological features, and associated with a poor prognosis. Currently, there is no standardized treatment protocol for SMARCA4-dNSCLC, as it generally exhibits a poor response to conventional chemotherapy regimens. Furthermore, the absence of typical driver gene mutations in lung cancer renders it insensitive to targeted therapies. We report the case of a 49-year-old Asian male patient with stage IV non-small cell lung cancer exhibiting SMARCA4 deficiency, who demonstrated a rapid response following treatment with a combination of nab-Paclitaxel, cisplatin, and bevacizumab. However, the patient ultimately experienced treatment failure due to disease progression from brain metastases, resulting in an overall survival (OS) of approximately 12 months. This case demonstrates the complicated clinical features of SMARCA4-deficient lung cancer, enriches the limited relevant clinical literature, and provides supplementary clinical data for this rare tumor subtype. While patients with SMARCA4-deficient lung cancer may benefit from chemotherapy combined with anti-angiogenic targeted therapy, the overall prognosis remains poor.

## Introduction

SMARCA4-deficient lung cancer represents a rare and highly aggressive malignancy characterized by undifferentiated or sarcomatoid pathological morphology, rapid clinical progression, and a notably poor prognosis (1–4). The BRG1 protein, encoded by the SMARCA4 gene, is crucial for the regulation of chromatin structure, as well as cell differentiation and proliferation. Its absence can result in genomic instability and epigenetic abnormalities, thereby facilitating malignant transformation (5, 6). Tumors of this nature typically lack classical driver gene mutations, such as those in EGFR or ALK, and exhibit limited responsiveness to conventional targeted therapies (7, 8), leading to the absence of a standardized treatment protocol. This paper presents a case of a patient with stage IV SMARCA4-deficient lung undifferentiated cancer, with the objective of thoroughly investigating the molecular mechanisms, therapeutic challenges, and potential intervention strategies associated with this disease, thereby providing a valuable reference for clinical management. This case report adheres to the CARE (CAse REport) (9) guidelines to ensure comprehensiveness and transparency.

## Case presentation

A 49-year-old Asian male with a history of smoking and alcohol consumption presented with cough and fever on December 9, 2023. Prior to admission, chest computed tomography (CT) performed at an external facility revealed a mass in the right lung, accompanied by obstructive pneumonia. Following antipyretic and anti-infective treatment, the patient’s fever symptoms subsided. The patient was then transferred to our hospital for further management.

**Figure 1:**
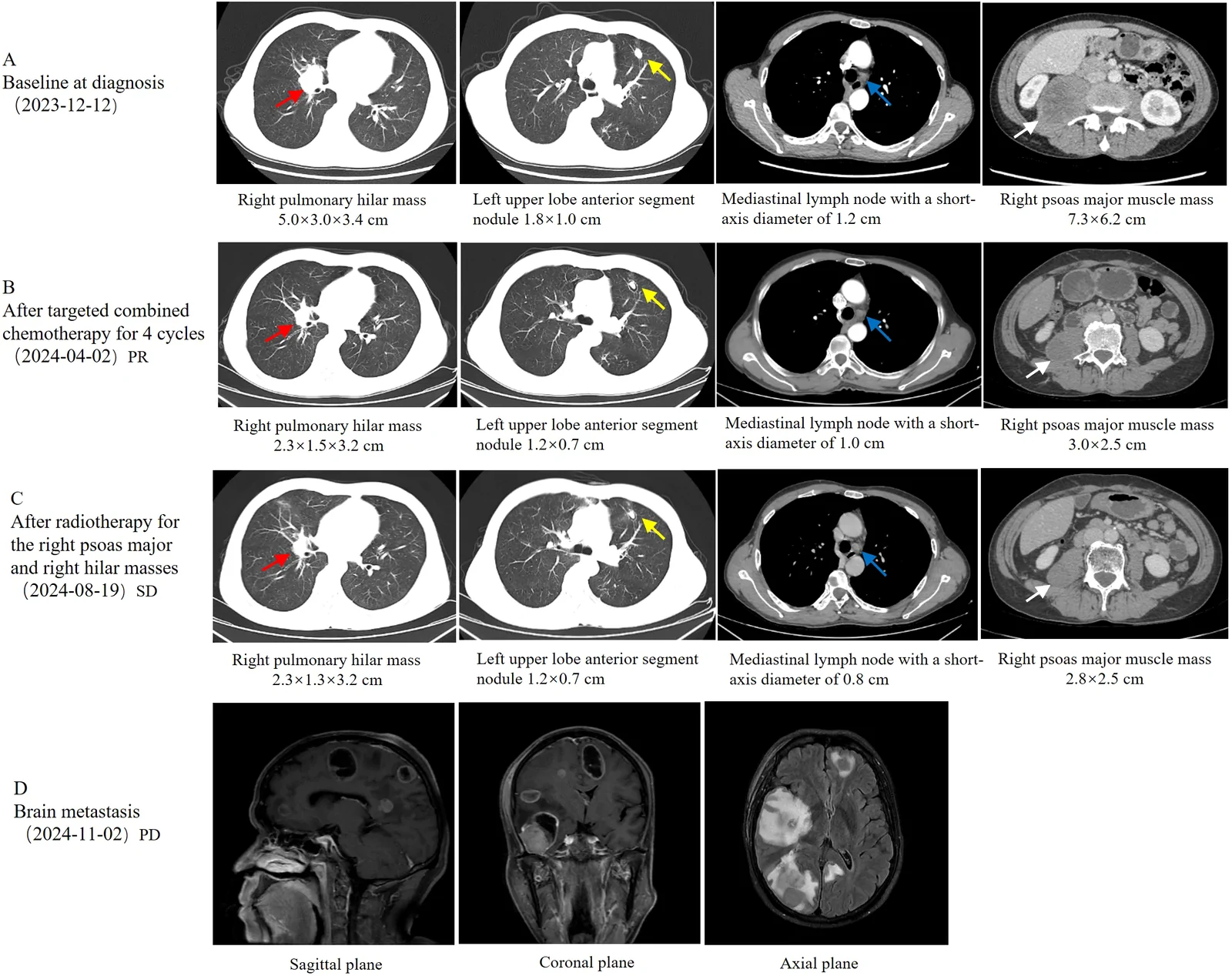
Imaging results. **(A-C)** Chest and abdominal CT images. Right pulmonary hilar mass (red arrow). Left upper lobe anterior segment nodule (yellow arrow). Mediastinal lymph node (blue arrow). Right psoas major muscle mass (white arrow). **(D)** Brain MRI showing multiple intracranial metastases.

**Figure 2:**
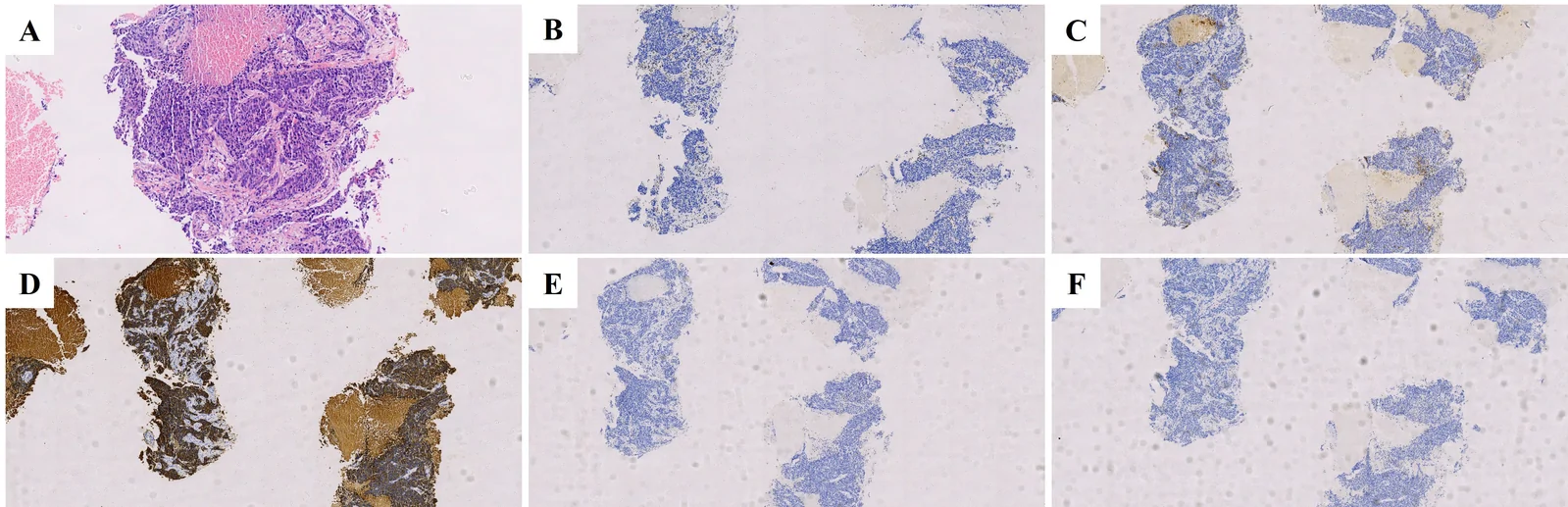
Pathological results. **(A)** Hematoxylin-eosin staining (×200) of the tumor. **(B-F)** Immunohistochemical staining of the tumor (×100): BRG1 (–), CK7 (+), CKP (+), TTF-1 (-), and Napsin A (–).

A transnasal bronchoscopy identified external compression resulting in the stenosis of the right middle lobe bronchus. The ThinPrep Cytology Test (TCT) analysis of the bronchoalveolar lavage fluid revealed a moderate presence of inflammatory and epithelial cells, with no evidence of malignant cells. On December 12, 2023, an enhanced chest CT scan indicated the presence of a potential tumor at the right pulmonary hilum, characterized by a well-defined, round mass measuring approximately 5.0 cm × 3.0 cm × 3.4 cm, and exhibiting mildly heterogeneous enhancement. The scan also revealed a 1.8 × 1.0 cm nodule in the anterior segment of the left upper lobe of the lung, along with several slightly enlarged mediastinal lymph nodes, and the enhancement was relatively uniform. The largest short-axis diameter was approximately 1.2cm. Subsequent abdominal CT imaging showed swelling of the right psoas major muscle, with a mass measuring approximately 7.3 cm × 6.2 cm, displaying clear boundaries and uneven enhancement post-contrast, suggesting a high likelihood of a tumor (**Figure 1A**). On December 18, 2023, an ultrasound-guided percutaneous biopsy was performed on a mass located in the right psoas muscle. Pathological analysis revealed a malignant tumor consistent with SMARCA4-deficient undifferentiated carcinoma. Immunohistochemical (IHC) evaluation demonstrated a loss of BRG1 expression, with CK(P) positive, focal positivity for CK7, CK5/6, and CD56, while CgA was negative. The Ki-67 proliferation index was over 70%, and there was partial positivity for Synaptophysin, while TTF-1 (SPT24), P40, NapsinA, and GATA-3 were negative. Additionally, there was partial positivity for P53, positivity for Rb, focal weak positivity for CDX2, and negativity for PAX8, Vimentin, and INI-1 (**Figure 2**). Subsequent diagnostic imaging, including cranial MRI and a whole-body bone scan, revealed no evidence of metastatic disease. The patient was unable to obtain pathological confirmation via percutaneous lung biopsy due to the presence of a lung mass. Following a multidisciplinary treatment (MDT) for lung cancer at our institution, it was concluded that the mass in the right lung represented the primary lesion. Consequently, the patient was diagnosed with a right hilar malignant tumor, classified as stage IVB (cT4N3M1c) with an Eastern Cooperative Oncology Group (ECOG) performance status score of 0.

**Figure 3:**
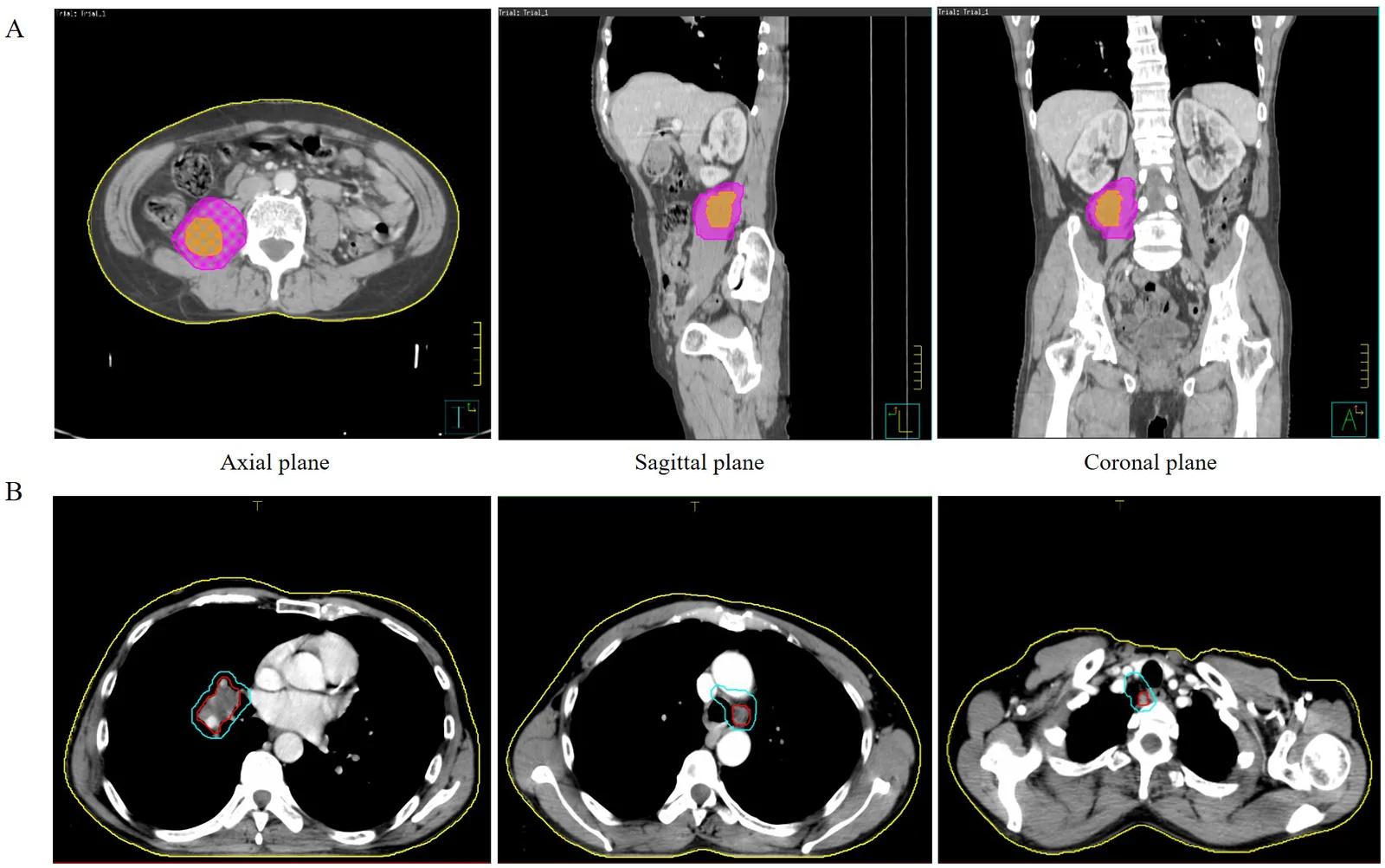
Radiotherapy target volume. **(A)** Radiotherapy target volume for a right-sided psoas major muscle metastasis. The dose was 75 Gy in the yellow part (GTV-core: necrosis area), 50 Gy in the purple part (pGTV: radiographically visible right psoas major muscle metastasis). **(B)** Radiotherapy target volume for the right hilar tumor and mediastinal metastatic lymph nodes. The dose was 60 Gy in the red part (GTV: radiographically apparent tumor), 54 Gy in the blue part (PTV: Planning target volume).

Owing to the patient’s financial limitations, genetic testing was not performed, resulting in an unknown PD-L1 expression status. Under the current health insurance reimbursement policy, PD-1 checkpoint inhibitors are not covered, whereas bevacizumab is eligible for reimbursement. According to the 2023 guidelines from the Chinese Society of Clinical Oncology (CSCO) for the diagnosis and treatment of non-small cell lung cancer, the combination of bevacizumab with platinum-based doublet chemotherapy, followed by bevacizumab maintenance therapy, is a class I recommended treatment protocol. Consequently, a treatment regimen was initiated on December 27, 2023, consisting of targeted therapy [bevacizumab 7.5 mg/kg every three weeks (10)] in combination with chemotherapy (nab-paclitaxel 260 mg/m² (11) and cisplatin 75 mg/m² every three weeks). Following four cycles of treatment, a follow-up chest and abdominal CT scan (**Figure 1B**) indicated a partial response (PR) in accordance with RECIST 1.1 criteria. Given the patient’s inability to tolerate chemotherapy due to Grade III bone marrow suppression, chemotherapy was discontinued. Between April 9, 2024, and May 11, 2024, the patient underwent radiotherapy targeting the metastasis in the right psoas muscle, with the treatment plan specified as follows: pGTV: 50 Gy over 25 fractions, GTV-core: 75 Gy over 25 fractions (**Figure 3A**). During this period, bevacizumab at a dose of 400 mg was administered as maintenance therapy. Subsequently, from June 14, 2024, to July 26, 2024, the patient received radiation therapy for the lesions in the right pulmonary hilum and associated metastatic lymph nodes, with the treatment plan outlined as: PTV: 54 Gy over 30 fractions, GTV: 60 Gy over 30 fractions (**Figure 3B**). Due to the tumor’s proximity to blood vessels, bevacizumab maintenance therapy was suspended during the radiation treatment.

**Figure 4:**
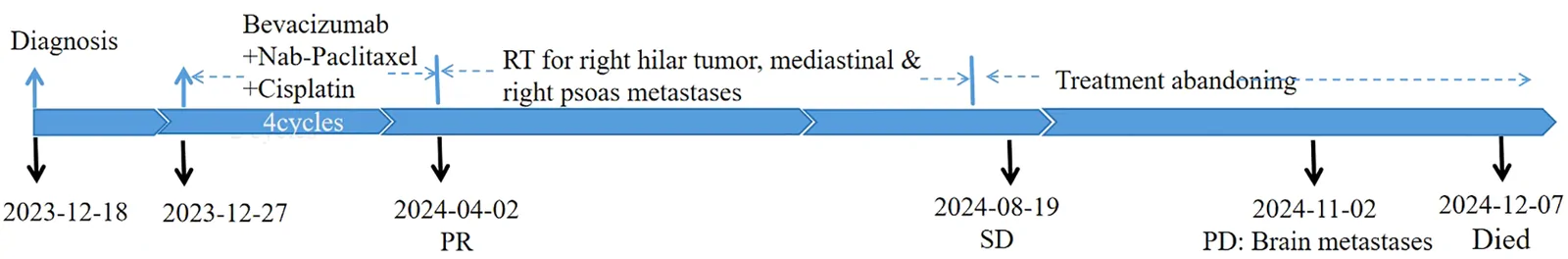
Case summary of disease progression and treatment outcomes: PR (partial response), SD (stable disease), PD (progressive disease).

Following the completion of radiotherapy for the patient’s pulmonary lesions, subsequent imaging (**Figure 1C**) demonstrated a stable disease (SD) condition, leading to the recommendation to continue maintenance therapy with Bevacizumab. However, financial limitations prevented the patient from pursuing this treatment. On November 2, 2024, the patient was admitted to the hospital with a headache, and a cranial MRI revealed multiple intracranial metastatic tumors (**Figure 1D**). The family decided to cease anti-tumor therapy, and the patient succumbed to the illness on December 7, 2024. A comprehensive overview of the disease progression and treatment regimen is depicted in **Figure 4**.

## Discussion

SMARCA4 is a critical component of the SWI/SNF chromatin remodeling complex, and its functional loss is strongly correlated with aggressive phenotypes and poor prognosis across various malignancies (5, 6). In recent years, SMARCA4-deficient lung cancer has garnered significant attention due to its distinct molecular characteristics and clinical behavior. This article presents a case study of a patient with stage IV non-small cell lung cancer exhibiting SMARCA4 deletion, who initially demonstrated a rapid response to a treatment regimen comprising nab-paclitaxel, cisplatin, and bevacizumab. However, the treatment ultimately failed due to disease progression resulting from brain metastasis, with the patient achieving an OS of nearly 12 months. This case highlights the clinical challenges associated with SMARCA4-deficient lung cancer and offers valuable insights for optimizing therapeutic strategies for such patients.

The BRG1 protein, encoded by the SMARCA4 gene, plays a crucial role in modulating gene transcription through the regulation of chromatin structure. Its absence can result in genomic instability and epigenetic dysregulation, thereby facilitating tumorigenesis and metastasis (5, 12). In the context of lung cancer, the loss of SMARCA4 is frequently observed in pleomorphic carcinoma and certain non-small cell lung cancers, where it is strongly correlated with heightened invasiveness, resistance to chemotherapy, and accelerated disease progression. Empirical evidence indicates that the majority of patients with SMARCA4-deficient lung cancer succumb within six months, exhibiting markedly reduced survival durations compared to patients with the wild-type gene (13, 14).

In the present case, due to technical and safety concerns, percutaneous transthoracic needle biopsy of the pulmonary lesion was not feasible. Consequently, a biopsy of the right psoas major muscle was performed. It is clinically reasonable and consistent with standard oncological practice to sample a metastatic site (the psoas muscle) as a surrogate diagnostic specimen. Following MDT discussion, the IHC profile revealed the following findings: positive staining for CK(P) confirmed an epithelial origin, negative expression of Napsin A and TTF-1 effectively ruled out pulmonary adenocarcinoma, negative P40 staining excluded pulmonary squamous cell carcinoma. Focal positivity for Syn raised the differential consideration of pulmonary large-cell neuroendocrine carcinoma (LCNEC); however, the negative CgA staining and the Ki-67 proliferation index below the typical threshold for LCNEC supported a diagnosis of undifferentiated carcinoma. The concurrent loss of BRG1 (SMARCA4) and INI-1 expression provided the diagnostic basis for SMARCA4-deficient undifferentiated carcinoma (SDUC). Radiologically, the lesions were confined to the lung and the right psoas muscle. Although the pulmonary lesion was not biopsied, the principle of “unifying diagnosis” (i.e., one disease explaining all findings) was applied. While hematogenous metastasis of primary lung cancer—particularly sarcomatoid or undifferentiated carcinoma—to skeletal muscle is rare, such occurrences have been documented in the literature (15, 16).Therefore, the final diagnosis was established as right lung malignancy with psoas muscle metastasis (cT4N3M1c, stage IVB). It is noteworthy that SMARCA4 loss is often accompanied by co-mutations in other oncogenic drivers, such as KRAS and TP53, which may further intensify the tumor’s malignant phenotype (13, 14). Despite the diagnostic clarity achieved through the psoas biopsy and IHC profiling, we acknowledge the inherent limitations of this case report. The lack of pathological verification from the primary pulmonary lesion, coupled with the unavailability of NGS data (including SMARCA4 mutation subtypes and TMB), restricted our ability to fully delineate the immunophenotypic landscape and predict the patient’s response to immune checkpoint inhibitors. Nevertheless, this case underscores the diagnostic utility of surrogate site biopsy in clinically challenging scenarios and highlights the need for further molecular characterization in SMARCA4-deficient tumors.

Immunotherapy combined with chemotherapy has emerged as a cornerstone first-line treatment for advanced NSCLC. Studies have shown that patients with SMARCA4-mutant NSCLC may derive a superior median OS benefit from chemo-immunotherapy compared to chemotherapy (2). But in this case, due to the lack of genetic test results, the medical insurance cannot cover the cost of the PD-1 inhibitor, a treatment regimen comprising nab-paclitaxel, cisplatin, and bevacizumab was employed, resulting in a rapid PR following therapy. This outcome may be attributed to the synergistic effects of multiple mechanisms. Albumin-bound paclitaxel facilitates targeted delivery by binding to the SPARC protein secreted by tumor cells, thereby achieving higher intratumoral drug concentrations and reduced systemic toxicity compared to conventional paclitaxel (17–20). Cisplatin promotes apoptosis in tumor cells through the formation of DNA adducts, complementing the microtubule-stabilizing action of paclitaxel (21, 22). Additionally, bevacizumab, an anti-vascular endothelial growth factor (VEGF) monoclonal antibody, inhibits tumor angiogenesis, remodels the tumor microenvironment, and enhances the permeability of chemotherapeutic agents (23). Previous studies have demonstrated that combining angiogenesis-inhibiting drugs with chemotherapy can significantly extend progression-free survival (PFS) in patients with advanced non-small cell lung cancer (24, 25). The prompt therapeutic response observed in this case indicates that, although SMARCA4 deficiency commonly confers inherent chemoresistance, combinatorial regimens pairing cytotoxic chemotherapy with anti-angiogenic agents can partially reverse the adverse molecular prognostic drivers underlying this malignancy.

Despite the patient’s initial positive response to treatment, ultimate treatment failure was observed due to the progression of brain metastases. This occurrence underscores the propensity of SMARCA4-deficient lung cancer to metastasize to the central nervous system, consistent with its highly aggressive nature. Moreover, the blood-brain barrier may impede the effective penetration of chemotherapy agents and bevacizumab into the central nervous system, rendering the brain a ‘sanctuary’ for tumor cells. The pronounced invasiveness and metastatic potential of SMARCA4-deficient lung cancer demonstrate malignant biological behavior akin to that of small cell lung cancer. In this instance, the patient did not undergo prophylactic cranial irradiation or receive blood-brain barrier-penetrating targeted therapy following stabilization with systemic treatment, potentially hastening the clinical manifestation of brain metastases. This treatment failure highlights the necessity for early evaluation of central nervous system metastasis risk in patients with SMARCA4-deficient lung cancer and advocates for the incorporation of local therapies, such as stereotactic radiation, to enhance prognosis.

While the OS of patients extends to approximately 12 months—surpassing the anticipated survival rate for most individuals with SMARCA4-deficient lung cancer—the survival advantage is predominantly observed during the initial phase of treatment. The duration of survival markedly diminishes following the onset of brain metastasis, underscoring the critical need for a comprehensive management strategy for SMARCA4-deficient lung cancer. Future investigations could examine the potential implementation of maintenance therapy, such as monotherapy with Bevacizumab, subsequent to an intensified combination treatment. Additionally, the feasibility of prophylactic cranial irradiation or the early introduction of immune checkpoint inhibitors, like PD-1/PD-L1 inhibitors, warrants exploration to potentially activate the immune microenvironment within the central nervous system and thereby enhance therapeutic efficacy.

The therapeutic challenge posed by SMARCA4-deficient lung cancer necessitates personalized approaches guided by molecular characteristics. Preclinical research indicates that SMARCA4 deficiency may result in compensatory activation of the EZH2 methyltransferase (26), suggesting that EZH2 inhibitors could selectively target SMARCA4-deficient cells through synthetic lethality, thereby serving as potential targeted therapeutic agents. Based on the paralog relationship between SMARCA2 and SMARCA4 within the SWI/SNF complex, SMARCA4-deficient tumors exhibit synthetic lethal dependence on SMARCA2, providing a rationale for developing SMARCA2 ATPase inhibitors and PROTAC degraders (27). Additionally, SMARCA4-deficient cells, characterized by intrinsically elevated DNA replication stress, are highly sensitive to ATR inhibition, and combination with PARP inhibitors exerts synergistic cytotoxicity, as validated in lung adenocarcinoma xenograft models (28). SMARCA4 deficiency has also been associated with upregulation of the oxidative phosphorylation pathway, and OXPHOS inhibition showed therapeutic potential in clear cell renal cell carcinoma models (29). Other studies have suggested links between SMARCA4 deficiency and cell cycle aberrations involving the CDK4/6 pathway (30), as well as G-quadruplex-mediated genomic instability that may be targetable by DNA damage response strategies (31). However, the vast majority of these findings remain at the *in vitro* or animal model stage and have not yet translated into clinical validation. At the early clinical level, SMARCA2 inhibitors (LY4050784) and degraders (PRT3789, PRT7732) have entered Phase I trials in SMARCA4-mutant solid tumors (NCT06561685, NCT05639751, NCT06560645); EZH2 inhibitor–immunotherapy combinations are also under investigation in Phase I/II studies, though efficacy data are pending. At the established clinical level, no targeted regimen has been approved for SMARCA4-deficient tumors.

Furthermore, these tumors frequently exhibit a high tumor mutation burden and elevated PD-L1 expression, suggesting that immunotherapy could be beneficial; however, caution is warranted due to the potential risk of hyperprogression. Lastly, optimizing treatment strategies for brain metastases, such as employing small molecule tyrosine kinase inhibitors or nanoparticle drug carriers to penetrate the blood-brain barrier, may enhance control of central nervous system involvement.

## Conclusion

This case highlights the clinical intricacies associated with SMARCA4-deficient lung cancer. While multimodal combination therapy has the potential to induce swift remission, the challenges of brain metastases and therapeutic resistance continue to impede advancements in patient survival. Future strategies should focus on the integration of molecular profiling, localized treatment modalities, and novel targeted therapeutics to develop a personalized treatment framework informed by dynamic risk assessment. Concurrently, translational research into the biological mechanisms underlying SMARCA4 deficiency is imperative to furnish essential scientific insights necessary for overcoming existing therapeutic limitations.

## Patient perspective

The patient was a farmer with limited financial resources. When diagnosed, he felt anxious and even considered giving up treatment. However, the doctor devised a treatment plan for him where some of the medications could be covered by the medical insurance, thereby reducing the financial burden. After the patient’s treatment, the cough symptoms significantly improved. The imaging examination results after two cycles indicated that the treatment was effective, providing additional information for his subsequent treatment.

## Data Availability

The original contributions presented in the study are included in the article/**Supplementary Material**. Further inquiries can be directed to the corresponding authors.
